# Phages and Their Role in Gastrointestinal Disease: Focus on Inflammatory Bowel Disease

**DOI:** 10.3390/cells9041013

**Published:** 2020-04-18

**Authors:** Martin Maronek, Rene Link, Lubos Ambro, Roman Gardlik

**Affiliations:** 1 Institute of Molecular Biomedicine, Faculty of Medicine, Comenius University, 811 08 Bratislava, Slovakia; martin.maronek@gmail.com; 2 Institute of Experimental Medicine, Faculty of Medicine, University of Pavol Jozef Šafárik, 040 11 Košice, Slovakia; rlink4@gmail.com (R.L.); lubos.ambro@upjs.sk (L.A.)

**Keywords:** virome, phageome, inflammatory bowel disease, intestinal inflammation, phage therapy

## Abstract

Inflammatory bowel diseases (IBDs) are a group of chronic autoinflammatory diseases including Crohn’s disease and ulcerative colitis. Although the molecular mechanisms governing the pathogenesis of gastrointestinal inflammation are not completely clear, the main factors are presumed to be genetic predisposition, environmental exposure, and the intestinal microbiome. Hitherto, most of the studies focusing on the role of the microbiome studied the action and effect of bacteria. However, the intestinal microbiome comprises other members of the microbial community as well, namely, fungi, protozoa, and viruses. We believe that bacteriophages are among the main orchestrators of the effect of microbiota on the gut mucosa. Therefore, this review aims to summarize the knowledge of the role of intestinal phageome in IBD and to discuss the concept of phage therapy and its future applications.

## 1. Introduction

The human body harbors a vast and complex ecosystem comprising microbes, bacteria, fungi, viruses, and other living organisms, collectively known as the microbiome. It is commonly present on the skin, urogenital, and respiratory tracts; however, the most abundant microbial populations can be found in the lower part of the gastrointestinal tract, the colon. The part of the microbiome residing in the small intestine and the colon (here termed the “intestinal microbiome”) as a whole bestows a multitude of effects upon the host ranging from modulating digestion and food passage to production of vitamins, short-chain fatty acids, and various other metabolites, as well as education of the immune system or helping to maintain a balanced state [1].

Given that the gastrointestinal tract is the major line of microbe–host interactions with the highest microbiota abundance in the body, the intestinal microbiome was clearly primarily studied from the view of gastrointestinal disease. Inflammatory bowel diseases (IBDs) are a group of chronic autoinflammatory diseases including ulcerative colitis (UC) and Crohn’s disease (CD). They are characterized by a long-lasting recurrent inflammation of various parts of the gastrointestinal tract. Epidemiological data show that the highest prevalence worldwide is documented in Europe, where Norway (505 UC patients per 100,000 people) and Germany (322 CD patients per 100,000 people) are the leading countries [2]. Previously, it was thought that IBD occurs mainly in Western developed countries, probably owing to lifestyle habits. However, in recent decades, the trend shifted toward developing and newly industrialized countries in Asia, Africa, and South America. This means that IBD presents a considerable burden for the healthcare system of many Western countries and will probably affect the remainder of the world in the near future [3].

Although there is currently a large variety of available therapies, none of them offer complete remission to every patient. Moreover, frequent cases of treatment ineffectiveness are being reported, forcing patients to either change the medication or increase the dosage [4]. High dosage of any medication carries the risk of worsening any potential side effects. With this in mind, finding new approaches to treat chronic inflammation in IBD is imperative. As such, fecal microbiota transplantation (FMT) and phage therapy (PT) are being considered.

## 2. Role of Dysbiosis in Disease

It is estimated that an average human colon contains approximately 10^11^ bacterial cells per one gram of fecal content [5,6,7]. More recent studies estimated the number to be approximately 10^12^ bacteria on the skin and 10^13^ in the intestine. This huge amount is said to outnumber the cells of our own body by a factor of 10 and continues to be frequently cited even in present day [8]. More recently, however, this estimate was challenged by Sender et al. They proposed a ratio of only 1.3:1 bacterial to human cells on the basis of certain assumptions for an average male [9]. As viruses and virus-like particles are, in general, much smaller than prokaryotes, estimates for their total abundance in the human gut may vary even more widely than those of the bacteria.

The balance of the gut microbiome is a key feature. The composition of the intestinal microbiome is not static in time. It undergoes constant changes that depend on various factors, such as overall health state, environmental exposure (e.g., diet composition, smoking or exercise), and the local environment surrounding an individual [10]. If the composition of the intestinal microbiome undergoes massive qualitative and quantitative changes and the balance is impaired, then a dysbiotic state occurs. Dysbiosis has a negative influence on the health of a person, as it is associated with many even seemingly unrelated diseases including liver disease [11], neurobehavioral dysfunction in non-alcoholic steatosis [12], lung cancer [13], or obstructive sleep apnea [14]. Furthermore, dysbiosis may provoke the development or exacerbate the course of inflammatory diseases such as rheumatoid arthritis [15], systemic lupus erythematosus [16], or IBD [17].

The term “dysbiosis” usually encompasses changes in the abundance of intestinal microbiota. However, as bacteria are not the only residents of the human gut, bacterial dysbiosis very likely also affects viral composition and abundance. Bacterial and viral dysbiosis may possibly play a role in the development and progression of various diseases. In fact, viruses infecting prokaryotic cells, or bacteriophages (phages for short), are being associated with metabolic disorders such as type 1 diabetes [18], type 2 diabetes [19], or neurodegenerative diseases such as Parkinson’s disease [20].

## 3. Composition of Virome in Health and Disease

The analysis of the composition of each component of the microbiome is the first important step in understanding the interactions happening among viruses, fungi, bacteria, and the immune system of the host. Generally, viruses can be divided on the basis of their host preference into eukaryotic and prokaryotic. Although eukaryotic viruses that infect humans are probably the most widely studied, their number is low in proportion to the number of prokaryotic viruses. In fact, 90% of all viruses are phages, with the remaining 10% being plant and animal viruses [21].

One of the first reports regarding the composition of viruses in gut samples came from Reyes et al. They analyzed the composition of the fecal virome in adult female monozygotic twins and their mothers throughout a period of one year. They also found that, while the composition of the fecal bacteriome showed a higher degree of similarity compared with unrelated individuals, viromes were unique to an individual regardless of any genetic relatedness. Moreover, despite high interpersonal variability, intrapersonal differences remained low [22]. Subsequently, Pérez-Brocal et al. described the viral and microbial composition in fecal samples from CD patients and controls. Viral families *Siphoviridae*, *Myoviridae*, and *Podoviridae*, among others belonging to the order *Caudovirales*, were found to be most abundant [23]. This research group later continued with the assessment of bacterial and viral populations in samples from CD patients in various stages of the disease in comparison with control individuals. Interestingly, the impact of individual variability and sample origin on the composition of viral communities was larger than the presence of CD. On the basis of these findings, the authors concluded that bacteria reflect disease status in a more accurate manner than viruses [24]. Contrary to what may be expected, mucosal samples from CD patients had a higher viral count compared with healthy individuals. By contrast, ulcerated mucosa showed a lower amount of phages than the healthy non-ulcerated intestinal tissue [25].

To date, studies fail to provide a definite answer, as some suggested that no differences exist between ulcerated and non-ulcerated mucosa [26] while others showed less bacteria in ulcerated mucosa [27]. Therefore, whether healthy or ulcerated mucosa is inhabited by more bacteria and the exact role of phages in their abundance are unclear.

Wagner et al. amplified viral sequences from ileal and colonic biopsies from pediatric CD patients and found the most viral hits in CD ileal samples followed by control ileal and CD colonic samples. The majority of viral reads belonged to *Caudovirales* [28]. A study by Norman et al. confirmed the expansion of this order in CD and UC patients. They speculated that the virome may contribute to intestinal inflammation and dysbiosis, and that it may play a role in other diseases [29]. Additionally, these results were supported by a T-cell transfer model of colitis in mice. In animals treated with T cells, reads mapped to the families *Siphoviridae*, *Podoviridae*, and *Myoviridae* were more elevated compared with control mice. However, the authors also stated that reads mapped to the genome of *Caudovirales* accounted for less than 0.01% of the total viral reads [30]. Consistent with these findings, *Caudovirales* phages were more abundant in CD than UC pediatric samples, although the same study did not find any difference between CD and controls [31]. The analysis of the rectal mucosa of UC patients revealed an expansion of *Caudovirales*. However, a decrease in diversity, richness, and evenness of this order in comparison with healthy controls was also observed. The study demonstrated that the mucosal virome might be substantially altered in UC patients [32].

Tokarz et al. recently published an analysis of the stool virome of pediatric UC patients. Their findings contradicted the accepted notion of the association of viruses with UC. Despite discovering a significantly higher occurrence of anelloviruses in the UC cohort compared with controls, they did not find any viral taxa that could be implicated in the onset of UC. This led to the conclusion that the presence of viruses in stool was not associated with the onset of UC [33]. However, *Caudovirales* seem to be important in the pathogenesis of *Clostridium difficile* infection (CDI). In patients suffering from CDI, higher abundance but lower diversity of this order was detected in comparison with controls. Subsequent FMT treatment resulted in a decrease in the abundance of *Caudovirales* in CDI patients [34]. In the case of colorectal carcinoma (CRC), results are inconclusive. A study by Hannigan et al. did not find significant differences in Shannon diversity or viral richness between patients and healthy controls. Their findings suggested an indirect role for the virome by altering the bacterial community [35]. Conversely, viral dysbiosis is associated with early- and late-stage CRCs. It was reported that these associations were independent of tumor stage, lymph node metastases, or other clinical markers [36].

Relatively recently, metagenomic analyses began to uncover differences in the amount and abundance of viral sequences in stool samples from IBD patients. In one such study, patients who were found to have sequences from family *Herpesviridae* showed an increased diversity of their microbiome [37]. Another study described a higher amount of *Hepadnaviridae* transcripts in UC patients compared with CD patients and controls. In the study, the samples from early-diagnosed treatment-naive IBD patients were used. Patients suffering from CD showed increased abundance of *Hepeviridae* compared with controls. The gut mucosa of UC samples was characterized by the lower concentration of *Polydnaviridae* and *Tymoviridae*, while the CD gut mucosa had reduced abundance of *Virgaviridae* [38].

Several caveats should be considered when assessing the usefulness of metagenomic studies. Firstly, there is still no widely accepted consensus regarding the protocol of sample collection or data evaluation. This fact contributes to the variability of the results, which makes the comparison of studies in this area more difficult. Secondly, in their study, Wang et al. mentioned that approximately one in four reads was unclassified [37]. The ratios reported by other studies vary considerably, but many viral sequences must apparently be characterized in order to obtain a complete picture. However, higher abundance of a certain taxon does not have to mean a direct causal relationship with any disease. Indeed, there is still much to be unraveled. Accordingly, our current understanding of virome in health and disease may be biased. Therefore, additional experiments will have to be undertaken to assess the precise role of virome in the development and progression of diseases, including IBD.

## 4. Phage Life Cycle in IBD

Every organism capable of reproduction (either via division, mating, or, in the case of viruses, using their hosts) is under evolutionary pressure. Because of this pressure, animals constantly try to increase their fitness and outcompete their competitors, usually by gaining food resources, shelter possibilities, or number of offspring. Since viruses and bacteria are one of the oldest forms of life on Earth, there is an ancient competition among them, probably as old as life itself. Due to the occurrence of mutations in each new generation, a few bacteria escape their viral attackers on every occasion. However, this process works vice versa as well; in each new generation, a few viral particles are slightly different from the rest, which means that even the mutated bacteria may not be completely protected against these viruses. Therefore, this constant struggle may resemble an “arms race” where both sides are doing their best to outcompete their opponent [39].

**Figure 1:**
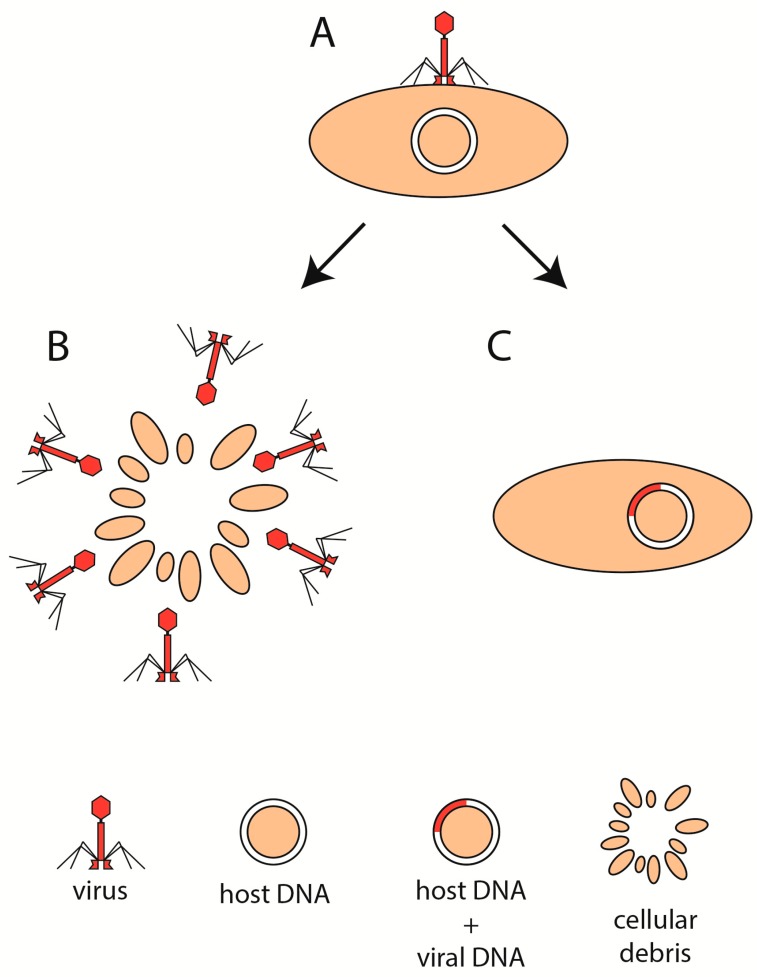
Phage life cycle. Following the attachment of virion to the host cell (**A**), the virus enters the lytic (**B**) or the lysogenic (**C**) state.

The life cycle of phages is undoubtedly one of the key features that shape the composition and abundance of gut bacteria at any given time. Generally, viruses can replicate in two ways (Figure 1A). They can use the proteosynthesis of infected cells to create many copies of its nucleic acid and subsequently release them to the environment via cell rupture, a process known as the lytic cycle (Figure 1B). However, certain viruses are able to incorporate their genetic information directly into the genome of the cell, ensuring that, after the next division, each daughter cell will carry, in its genome, the viral sequence as well. This process is called lysogeny and does not kill the cell instantly (Figure 1C). In this case, usually, only a few copies of the viral genetic information are being synthesized at any given time.

On the basis of the condition of the surrounding environment, phages constantly assess the probability of their success in reproduction via the fitness of their bacterial host. Some hypotheses provided explanations on the basis of experimental data. For instance, Clooney et al. hypothesized that one of the conditions contributing to a healthy colon is the presence of a “healthy” core virome [40]. They performed a whole-virome analysis on two IBD virome datasets, one being an in-house UC cohort and the other being a dataset published by Norman et al. [29]. The healthy core virome was absent in IBD samples. Moreover, it seems that IBD may be accompanied by a shift in the core virome from a virulent (lytic) life cycle toward temperate (lysogenic). The results are in line with another study where a stable core virome in healthy individuals was observed [41]. Thus, a scenario in which lytic phages help maintain a healthy gut environment is proposed.

Contrarily, there is also the “Piggyback the Winner” model in which phages may undergo the lytic cycle to take advantage of the rapid reproduction of bacteria. The model presumes that there is a gradient of lysogenic to lytic replication across the mucus layer of the intestine based on the amount of bacterial load in various parts of the mucosal layer [42,43]. Future research will likely uncover which of these hypotheses is closer to the truth. For now, however, the paucity of experimental data prevents us from grasping the role of immunity in phage–disease interactions.

## 5. Phage Therapy

The term “phage therapy” refers to the modulation of phageome and subsequently bacteriome of a person suffering from a disease which is thought to stem from bacterial origin. Usually, it involves several steps: modification of the genetic information of an existing phage in a way which ensures successful adsorption to the desired bacterial strain; preparation of either one or more phage strains; creation of a dosing schedule and administration of the phage preparation to the patient.

Although PT may seem a recent idea, mainly due to increasing antibiotic resistance, it was proposed back in the 1920s when Felix d’Hérelle cured patients suffering from diseases, such as dysentery or cholera [44]. However, the discovery and usage of antibiotics decades later overshadowed PT. Even today, antibiotic resistance is the main reason why PT is becoming viewed as a plausible alternative approach [45,46]. In the context of IBD and the crucial role of the gut microbiome in its pathogenesis, PT is considered a potential tool for microbiome modification via, more or less, the selective destruction of bacterial cells. Furthermore, however, there are two possible ways via which phages can shape the host and its response to their presence. Immune response to the phage particle components represents direct action of the host. Horizontal gene transfer from phages to bacteria represents indirect action.

## 6. Phage Immunogenicity

**Figure 2:**
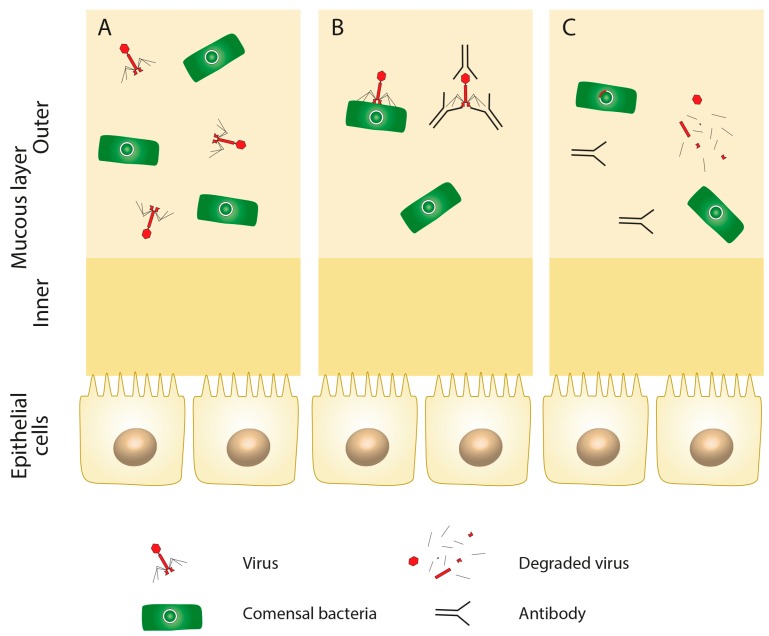
Phage immunogenicity. Even in the healthy intestine (**A**), some phages may elicit the immune response of the human host, which may lead to antibody formation (**B**) and the degradation of the viral particle (**C**).

An important aspect of possible PT for IBD that must be addressed is whether phages activate the immune system of the human host in a way which could cause inappropriate immune responses. Any phage engineered to be usable for PT should not be recognized by the immune system of the human organism. Several studies documented the ability of phages to stimulate the production of antibodies (Figure 2A–C) [47]. Interestingly, despite the presence of antibodies and a high rate of phage inactivation, PT may still bestow the desired effect [48].

In one study, the phage treatment of germ-free mice led to the expansion of immune cells in the intestine. It was reported that *Lactobacillus*, *Escherichia*, and *Bacteroides* phages stimulated interferon-γ (IFN-γ) via Toll-like receptor 9, a member of the pattern recognition receptors, which are the mediators of innate immunity. Moreover, UC patients responsive to FMT had a reduced number of phages compared with non-responders, while mucosal IFN-γ positively correlated with the concentration of phages. It was also found that phages from active UC patients induced a higher IFN-γ response compared with controls [49].

On the basis of these findings, it could seem that phages affect the environment of the gut negatively. However, there is evidence that they may also act in an anti-inflammatory manner. In one study, the T4 phage was immunomodulatory by reducing the production of reactive oxygen species (ROS). In addition, this phage inhibited ROS generation from peripheral blood polymorphonuclear leukocytes stimulated with either lipopolysaccharides or strains of *Escherichia coli* [50]. Górski et al. observed that the T4 phage decreased the infiltration of immune cells to an allogeneic skin transplant and lowered the proliferation of T cells and nuclear factor kappa B (NF-κB) activation in murine models [51].

**Figure 3:**
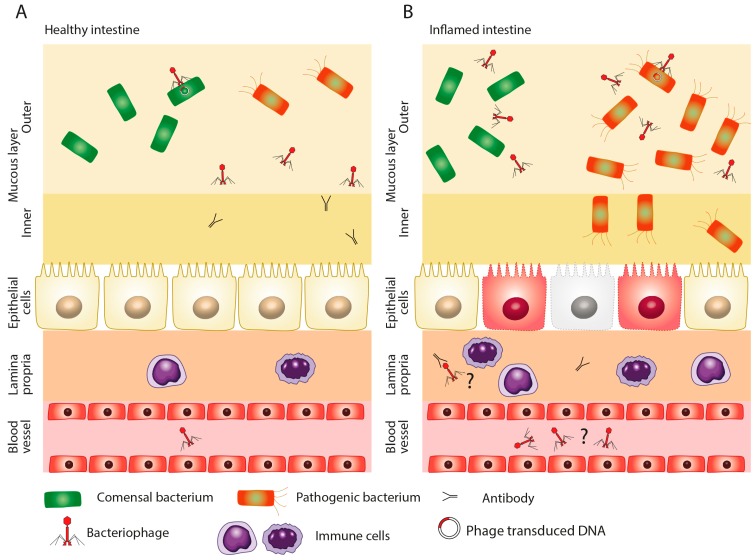
Presence of phages in circulation. Phages may be present in the circulation in a healthy individual (**A**) as well as during the course of intestinal inflammation (**B**). The higher abundance of phages in the circulation during intestinal inflammation may be due to increased intestinal permeability and the erosion of the mucosal wall.

Relatively recently, it was discovered that NF-κB activity can be modulated by *Staphylococcus aureus* phage [52]. In fact, the systemic presence of phages in the human body could play an important role in dampening the immune response and the development of autoinflammatory or inflammatory disorders such as IBD [53]. According to the results of Górski et al., the phages can possibly cross the mucosa and enter the systemic circulation even in healthy individuals [51]. This transition could be immunomodulatory. Unsurprisingly, when the mucosal wall is damaged during inflammation and intestinal permeability increases, so does the number of viral particles in the circulation (Figure 3A,B). However, the mechanisms of phage immunity modulation still elude our understanding [54]. In addition, it should not be forgotten that the rapid lysis of bacterial cells via the action of lytic phages may lead to overaccumulation of proteins, lipopolysaccharides, and nucleic acids, which could potentially fuel the inflammation even more [29].

**Figure 4:**
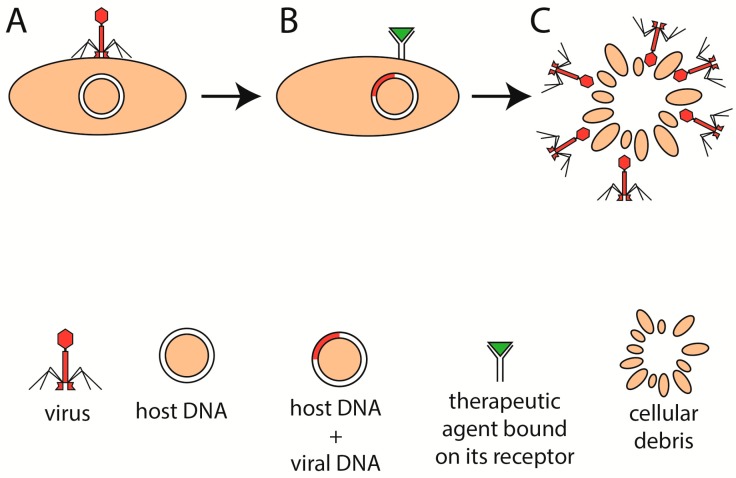
Modulation of phage therapy. The phage attaches to its host and incorporates its genetic information into the DNA of the host (**A**). If the bacterium was to be destroyed, for example, because of dysbiosis or pathogenicity, then the administration of a therapeutic agent (**B**) into the colon might induce the lytic cycle of the incorporated phages and subsequently degrade the bacterium (**C**).

There is a lack of experimental data regarding whether phage immunogenicity is related to the phage life cycle. Virulent and lysogenic life cycles have their advantages and reasons to be used. One might argue that introducing a preparation of several phages (referred to as a “phage cocktail”) into the colon of an IBD patient that could not incorporate its genetic information into bacteria may be important. Lysogenic phages might not destroy the desired bacterial strain and, thus, be so efficient in comparison with lytic phages. Therefore, phages for IBD patients may be engineered to lack integrase, the enzyme responsible for genome integration [55]. However, it may be even more useful if the ability to switch between lytic and lysogenic life cycles could be controlled (Figure 4A–C). It might be beneficial if the phage stayed incorporated in the genome of the bacteria of the host (otherwise, it would get depleted from the colon eventually), ready to strike if the balance of gut microbiota shifted toward a dysbiotic state. With these and many more questions in mind, how important the aspect of the phage life cycle will be for the efficiency of PT remains to be found.

## 7. Phages and Horizontal Gene Transfer

**Figure 5:**
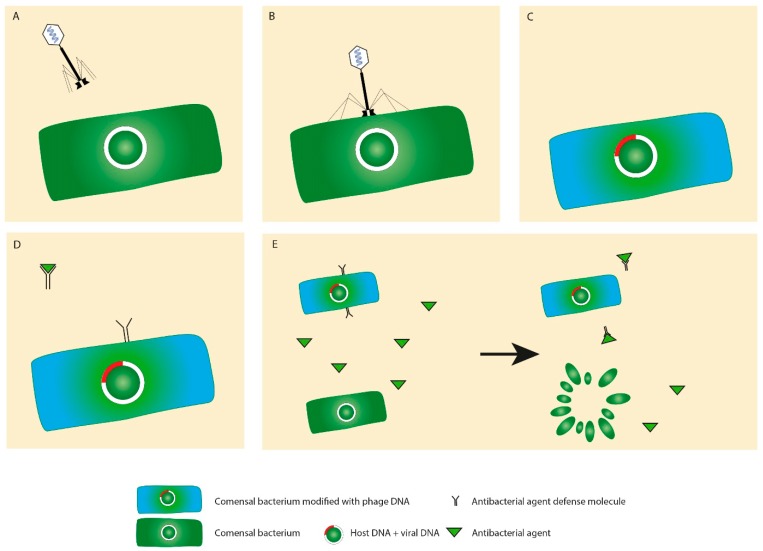
Horizontal gene transfer. In the process of viral infection (**A**,**B**), bacteria may be equipped with additional genes (**C**), such as genes coding for antibiotic resistance (**D**) or increased pathogenicity that may increase the fitness of the transformed bacterium in contrast to a bacterium that does not have this advantage and, thus, is destroyed (**E**).

In addition to the immunogenicity, phages may provide bacteria via horizontal gene transfer with additional genetic material (Figure 5A–E). Such genes may code toxins and products facilitating, for example, adhesion to the mucosa and invasion into the tissue or evasion from the immune cells of the host. For instance, a group of *E. coli* strains produce Shiga toxins, which originally came from phages [56]. Another example of phage-carried toxins is one of the most potent toxins, the botulinum neurotoxin [57]. In another study, the effect of four phages on the toxin production by *C. difficile* was examined. While none of the phages were able to convert the bacteria to produce the toxin, phage infection resulted in an increase in toxin B production [58].

*Vibrio cholerae*, the causative agent of cholera epidemics, has its main two virulence factors (cholera toxin and toxin coregulated pilus) of phage origin as well. These are encoded by phage CTXφ, and even other phages cooperating with CTXφ were characterized [59]. In addition, *Salmonella typhimurium* uses a specialized type III secretion system to translocate effector proteins into host cells. The effector proteins are conserved among *Salmonella* strains, and it is presumably a result of horizontal gene transfer. Moreover, phages carrying antibacterial resistance genes were detected in the sputum from cystic fibrosis patients [60]. Conversely, another study analyzed the abundance of antibiotic resistance genes in more than one thousand phage genomes. On the basis of the results, the authors concluded that the presence of antibiotic resistance genes in phages was vastly overestimated and is much more rare than previously thought [61].

Instead of acting directly, viruses may shape the effect of bacteria indirectly, for example, via lysogeny. Experimental data suggested that SopEPhi, a member of P2 family of phages, is able to transfer genes between different *S. typhimurium* strains by lysogenic conversion [62]. One such gene codes the SopE effector protein. This protein triggers mucosal inflammation via the activation of caspase-1 and the secretion of interleukin-1β [63]. Therefore, phage SopEPhi indirectly contributes to the development of colitis in streptomycin-pretreated mice [64]. Some proteins are even able to bind C5a, a component of the complement system, and inhibit the chemotaxis of neutrophils and monocytes. This category is represented by an exoprotein CHIPS (chemotaxis inhibitory protein of *S. aureus*) produced by *S. aureus* strains [65].

The putative contribution of bacteriophages to the regulation of the intestinal bacteria was previously reviewed by our group [66]. The mechanisms via which bacteriophages modulate the bacterial microbiota in the gut are multifactorial. Upon incorporation into pathogenic bacteria, the prophages carrying genes encoding antibiotic resistance may act as procolitic factors, but may also be anti-colitic when incorporated into probiotic bacteria. Moreover, stress-induced activation of a prophage might lead to activation of its lytic cycle and subsequent reduction of the number of the host bacteria. The disrupted environmental niche might then be replaced by pathogenic (or commensal) bacteria with procolitic (or anti-colitic) effects. In addition, due to the reduced mucosal layer thickness, the protective interactions between phages and mucosal glycoproteins may be damaged, paving the way for pathogenic bacteria.

## 8. Fecal Microbiota Transplantation as a Tool for Phage Therapy

FMT is effective in multiple gastrointestinal diseases such as CDI, irritable bowel syndrome (IBS), IBD, and CRC [67,68]. For instance, in the case of recurrent CDI, it reached the effectiveness of over 80% even after a single treatment [69]. There is a reason to believe that it could achieve similar efficiency in IBD as well. However, the exact composition of the transplant from a healthy donor in many cases remains undescribed.

One of the first studies focusing on the composition of the virome after FMT found the transfer of viral sequences from a healthy donor to pediatric UC patients. Among the sequences, the members of *Siphoviridae* were transferred with greater efficiency than other groups [70]. Of particular importance is also a study by Broecker et al. who found the phage population of a recipient CDI patient after FMT to be very similar to the donor. Unexpectedly, the phage composition remained stable for up to seven months. The composition of bacteria, in contrast, varied longer before reaching donor similarity [71]. Still, evidence of how the composition of the virome changes in time during various gastrointestinal diseases or how changes in the virome correspond to the severity of the disease is unavailable.

Although the efficiency of FMT extends even beyond gastrointestinal diseases [72], the majority of studies used freshly prepared fecal suspensions. These suspensions usually contain viable bacteria to achieve the desired effect. Until the work of Ott and colleagues, it was not known what would happen if the donor fecal matter was sterile instead. Using filters with a pore diameter of up to 0.2 μm, they removed any bacteria from the fecal suspension. Next, they administered the filtrate to five CDI patients. Interestingly, in all five patients, symptoms of CDI were eliminated, and the improved condition lasted for at least six months. Given that no living bacteria were present in the filtrates, the authors suggested the action of phages as a possible explanation [73]. This discovery opens a whole new area to explore. The question is whether such an FMT graft devoid of living bacteria is able to mediate the desired therapeutic effect, such as alleviation of IBD or other gastrointestinal diseases. Removing the necessity of the presence of living bacteria in FMT graft could also provide an option for immunocompromised patients.

Our previous findings suggest that the effect of bacterial therapy can be at least partially mediated by various parts of the bacterial cell, as even non-viable bacterial vectors showed certain therapeutic effect in IBD treatment [74]. The theory that the effect of FMT can be mediated not only by living bacteria is also supported by the hypothesis of reverse phenotype transfer by FMT that was recently supported by preliminary results [75]. Taken together, it seems that the beneficial effect of FMT may not be entirely conferred by living bacteria and other mechanisms might play crucial role, including phages present on the graft. However, there is not yet enough evidence to conclude that knowledge is applicable in IBD management.

## 9. Phages Versus Antibiotics

When considering the potential use of phages for therapeutic purposes, advantages and disadvantages compared with antibiotics must be addressed. Phages are of viral origin; therefore, they differ completely from antibiotics in many aspects. For instance, phages are usually strain-specific, whereas antibiotics are active against a wide range of bacterial species, often killing harmless commensals along with pathogens. This approach may, in turn, lead to other problems such as antibiotic-associated diarrhea and *C. difficile* infection [76]. Conversely, phages do not appear to inflict any undesired damage upon the microbiota. These assumptions are, however, based on our incomplete understanding of interactions occurring between phages and bacteria and should, thus, be studied in more depth.

Although PT may be a reliable option against an infection caused by a single bacterial strain, there are situations in which a multitude of phages may be needed. Creating a phage cocktail is possible; however, compared with antibiotics, this approach is much more challenging [77]. The specificity of phages is also a limiting feature in contrast to antibiotics when it comes to mass production. Another probably less-known constraint for phages may be their region-restricted efficiency. This knowledge comes from a study in which phages were more effective against diarrhea-associated *E. coli* of the same region [78]. In addition, bacteria are often present in a biofilm. This consists mostly of polysaccharides, proteins, and lipids that form together a net-like structure. The penetration of this layer presents a challenge for antibiotics. However, evolution endowed phages with various enzymes capable of biofilm degradation. Phages can, thus, infect bacteria that would otherwise remain unreachable [79,80]. Thus, phages possess advantages and disadvantages compared with antibiotics, and it would perhaps be unreasonable to try to think of PT as a complete substitution for antibiotics. Hence, there will always be cases in which antibiotics as a treatment of choice will be more effective and vice versa.

## 10. Safety and Efficiency of Phage Therapy

Any potential treatment must fulfill two basic criteria of being safe and causing fewer side effects and being more efficient, at least compared to any treatment already available. Research in this area was already conducted. For instance, the findings of a study by Galtier et al. look promising. Mice were infected with an adherent-invasive *E. coli* strain LF82 that was previously implicated in the pathogenesis of IBD [81,82,83]. After the isolation, purification, and sequencing of phages, phage preparations were administered to murine intestinal sections, living animals, and homogenates of ileal biopsies taken from CD patients. Across all the samples, phages significantly reduced the colony-forming units (CFU) of the *E. coli* LF82 strain. Phage treatment was even helpful in alleviating the symptoms of dextran sodium sulfate-induced colitis in mice colonized with *E. coli* LF82. Strikingly, this effect was achieved after only a single dose of phage preparation. On the basis of these results, the authors concluded that PT may be a suitable treatment option for CD patients infected with adherent-invasive *E. coli* strains [84].

Hitherto, multiple studies did not find any serious life-threatening adverse effects attributable to PT [85,86,87,88]. No safety concerns were observed in the phase I therapy of venous leg ulcers in humans [89]. Additionally, in a study where whole viral communities were transferred via FMT between human individuals, none of the transferred viruses were known to infect human cells [70]. On the basis of these results, phage usage appears to be safe without any serious side effects.

## 11. Future Perspectives

For decades, the gut microbiota as a potential modulator of the development and progression of diseases of the gastrointestinal tract including IBD was mostly overlooked by scientists. In recent years, the trend turned in favor of the intestinal microbiota; yet, from all the organisms that form the gut biosphere, bacteria are the main target of research. We believe that viruses, especially phages, deserve our attention as well. Although research in this field advanced considerably, there are still many challenges that must be resolved. For instance, it would be beneficial to see any potential changes in the composition of the intestinal phageome during the course of IBD.

So far, the majority of studies focusing on the composition of the virome in IBD identified the abundance at only a single time point. It might be interesting to see if changes in the composition of the virome during IBD correspond to the changes in the severity of the disease. In this regard, constructing a reference phageome of a healthy person could prove useful, and it could be viewed as another step bringing us closer to personalized medicine. Despite a growing body of research, experimental data are still relatively scarce in the field of the virome composition and effect in IBD, especially studies focusing on multiple time points. Along with any change in the severity of inflammation during remission or relapse, bacterial and viral populations likely adapt to the ever-changing conditions in the colonic environment.

While PT has the potential to become a reliable, safe, and effective treatment, there are hindrances that must be resolved. For instance, whether the lytic or lysogenic life cycle (or a combination of them) is the most suitable for the destruction of harmful bacterial strains and the alleviation of gastrointestinal disease, including IBD, must be determined.

In addition to the concern of safety, it must be established if the administration of a large dose of phages could not cause the excessive breakdown of bacteria in a short amount of time. This could lead to a high release of enterotoxins that could act as proinflammatory mediators and, thus, provoke overstimulation of the immune system. Moreover, the mere presence of phages and their antigens might represent a direct immunogenic factor for the intestinal immune system. How the immune system reacts to various phage-associated antigens systemically and locally in the gut was not sufficiently explored. Thus, more research is crucial to evaluate the impact of the virome on IBD, and whether this impact could provide grounds for future therapeutic implications remains to be found.

## 12. Conclusions

Phage-based approaches hold the potential to become a valid alternative option for the treatment of not only gastrointestinal diseases, including IBD. The results of present-day research suggest that the introduction of a phage cocktail via FMT or PT into the colon of patients suffering from gastrointestinal diseases is harmless and might provide a therapeutic benefit. However, mechanisms governing the key aspects, such as immunogenicity, horizontal gene transfer, or the composition of virome and phageome during flares and remissions, must continue to be studied to prove the potential. The complexity of the topic and the lack of hard evidence make it difficult to draw conclusions. In this regard, the creation of a standardized protocol for the isolation, detection, and quantification of viruses in the stool and intestinal biopsy samples and a reference panel for a healthy virome may prove helpful.
